# Blood Flow Restriction Training for Meniscus Repair Surgery

**DOI:** 10.1177/26350254231202532

**Published:** 2024-03-07

**Authors:** Yuki Yamanashi, Sachin Allahabadi, C. Benjamin Ma, Ivan Arriaga

**Affiliations:** † Department of Orthopaedic Surgery, Aichi Medical University, Nagakute, Japan; ‡ Department of Orthopaedic Surgery, Sports Medicine and Shoulder Surgery, University of California, San Francisco, San Francisco, California, USA; Investigation Performed at the Department of Orthopaedic Surgery, Sports Medicine and Shoulder Surgery, University of California, San Francisco, San Francisco, California, USA

**Keywords:** blood flow restriction, low-load training, meniscal repair, muscle atrophy, rehabilitation

## Abstract

**Background:**

Blood flow restriction (BFR) is a training tool that involves wearing a tourniquet or occlusive device during exercise. Data support that low-load training with BFR may produce muscle hypertrophy similar to standard high-load training. Because of the weight-bearing and range of motion (ROM) restrictions after meniscal repair, patients encounter substantial atrophy of lower extremity musculature. We perform BFR for these patients to limit atrophy postoperatively with the goal of facilitating their return to prior function and sports.

**Indications:**

We incorporate BFR in the postoperative rehabilitation protocol for patients undergoing meniscal repair not involving the root. Patients with the following are excluded: acute or severe cardiac disease, peripheral vascular disease, blood pressure over systolic 180 mm Hg or diastolic 100 mm Hg, hemophilia, thrombophlebitis or history of deep vein thrombosis, severe anemia, and sickle cell disease.

**Technique Description:**

An automated BFR device calculates the patient’s limb occlusion pressure (LOP) and titrates to 50% to 80% of LOP for lower extremity exercises. Exercise parameters typically consist of 4 sets of each exercise, totaling 75 repetitions, with 30-second interset rest. Patients undergo a standard 3-phase postoperative rehabilitation protocol. Phase I (weeks 0-6): Patients are nonweightbearing, may be either footflat weightbearing or partial weightbearing at the surgeon’s, with ROM restricted 0 to 90 in a hinge knee brace throughout the phase. Exercises include quadriceps sets with neuromuscular electrical stimulation and straight leg raises and short/long arcs quadriceps. Phase II (weeks 7-8): Patients progress to weightbearing and ROM as tolerated and begin exercises including double mini squats, hamstring curls, double leg press, and double leg heel raises. Phase III: (weeks 9+): Patients perform double and single leg bridges, double leg bridges on ball with knee band, squats, single leg press, and single leg heel raises, all with the goal of returning to sports.

**Results:**

Prior systematic review data demonstrate low-load training with BFR increases muscle strength and induces hypertrophy relative to low-load training alone. No significant differences for Knee Injury and Osteoarthritis Outcome Score (KOOS) subscales between BFR training group and control group.

**Discussion:**

BFR training may facilitate postoperative recovery in patients undergoing meniscal repair surgery by helping mitigate muscular atrophy.

**Patient Consent Disclosure Statement:**

The author(s) attests that consent has been obtained from any patient(s) appearing in this publication. If the individual may be identifiable, the author(s) has included a statement of release or other written form of approval from the patient(s) with this submission for publication.

## Graphical abstract

**Graphical Abstract:**
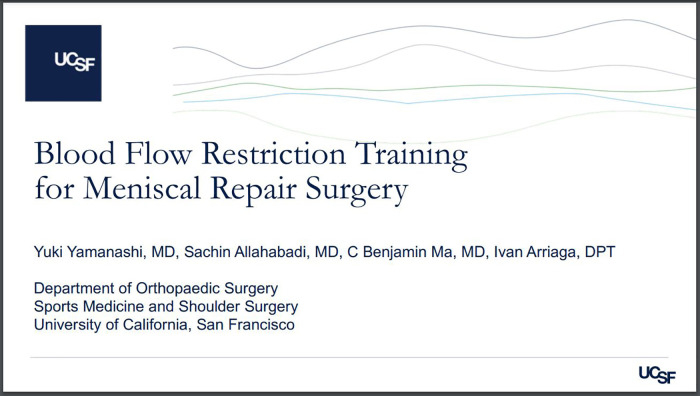
This is a visual representation of the abstract.

## Video Transcript

In this video, we will be reviewing the application of blood flow restriction (BFR) training following meniscal repair surgery.

Disclosures: Ivan Arriaga is an instructor for Bay Area Sports Performance and Rehabilitation, which hosts a continuing education course on clinical applications of BFR training. There are no products or services advertised during this publication.

BFR training is an exercise modality that involves wearing a tourniquet or occlusive device while performing exercise.

BFR training creates anaerobic conditions which upregulate anabolic pathways and protein synthesis. Moreover, it stimulates proliferation of myogenic cells and induces systemic hormonal responses favoring muscle recovery and growth.^7,9^

Supplementing BFR to low-load resistance training yields greater benefits in muscle strength and hypertrophy, with recent studies revealing a large effect size (standardized mean difference = 1.28) favoring improvement in muscle cross-sectional area and moderate effect size favoring strength increases (Hedges’*g* = 0.523, 95% confidence interval = 0.263-0.784, *P* < .001).^2,10^

Meniscal repair is a common surgical procedure following meniscal tears.

Weightbearing and range of motion (ROM) restrictions for often up to 6 weeks postoperatively lead to significant lower extremity musculature atrophy. A study by Kubota et al^4^ showed 2-weeks of nonweightbearing without BFR training caused a loss of 2.3% to 2.7% thigh circumference.

Preventing muscular atrophy and preserving muscle strength and function are critical goals of recovery for all patients, and especially those seeking to return to sport. However, early postoperative restrictions prevent these patients from performing traditional strength training. Therefore, these patients can greatly benefit from BFR implementation.

Prior studies on BFR have largely demonstrated improved outcomes after anterior cruciate ligament (ACL) reconstruction, knee osteoarthritis, sarcopenia, myositis, and other orthopedic conditions.^2,10^

These benefits may even extend proximally to the cuff and contralaterally.^1^ BFR training may begin within 1 week postoperatively. Patients with meniscus root repair may also benefit from BFR training but may require a more conservative protocol than the 1 proposed in this video.

Below is a list of contraindications to BFR to minimize risk of adverse effects. Previous studies have outlined potential concerns of BFR, including brain and petechial hemorrhage, rhabdomyolysis, deep venous thrombosis (DVT), venous injury, and induration.^8^ However, these risks are exceedingly rare and the most common risk is increased soreness in the musculature. Nevertheless, for safety, our contraindications include acute or severe cardiac disease, peripheral vascular disease, systolic blood pressure over 180 mm Hg or diastolic blood pressure over 100 mm Hg, hemophilia, thrombophlebitis or history of DVT, severe anemia, and sickle cell disease.

An automated BFR device is recommended to calculate and set a treatment occlusion pressure. In this example, it is the Delfi Personalized Tourniquet System.

A wide cuff is placed over the most proximal portion of the affected thigh. The BFR unit is then gradually inflates to calculate limb occlusion pressure (LOP), which is the total pressure required to completely occlude patient’s article blood flow to the lower extremity. It is critical to have the patient stay still and relaxed during this time for measurement accuracy.

The total LOP is displayed on top of the screen. The treatment occlusion pressure is then based off of a percentage of the total LOP, represented by the second number in blue. We are then able to set the BFR protocol occlusion pressure based on a percentage of the total LOP, represented by the second number in blue. In our protocol, we utilize between 50% and 80% of total occlusion pressure for lower extremity exercises, starting at the lower pressure and progressing toward, but never exceeding 80% over the first several visits according to patient tolerance. Patients are under occulsion for a maximum of 10 minutes continuosly. Once the pressure has been selected, the cuff is then inflated prior to beginning the exercises.

Our rehabilitation protocol has 3 phases at a frequency of twice to 3 times per week; 2 to 3 exercises are performed under BFR per session. Exercise parameters generally consist of 4 sets made up of 30-15-15-15 reps for each exercise with 30- to 60-second rests between sets. The BFR cuff remains inflated throughout all sets and rest periods of a single exercise and is deflated for a minimum of 1 minute between exercises to allow for reperfusion. BFR is also combined with neuromuscular electrical stimulation (NMES) during the first phase which utilizes unique parameters.

Phase 1 is postoperative 0 to 6 weeks. Patients typically restricted ROM and weightbearing precautions with a T Scope brace. The protocol first initiates quadriceps sets with NMES. BFR occlusion is applied for 4 minutes continuously followed by 1-minute of unoccluded rest for a total of 3 to 4 cycles. This totals 12 to 16 minutes under BFR occlusion. NMES to the quadriceps is applied throughout the entirety of the exercise with stimulation being applied for 8 seconds on and 3 seconds off. The patient actively engages their quadriceps muscle while the NMES is applied. Ankle pumps with a resistance band are then performed after the NMES and BFR protocol. After 2 weeks, the NMES and ankle pumps are discontinued and replaced with a combination of straight leg raises and short/long arcs quadriceps.

Phase 2 is postoperative weeks 7 to 8, which allows for weightbearing as tolerated and weaning from the T Scope brace. Before moving to phase 2, normal joint temperature and minimal to no joint effusion are required. Three BFR exercises are performed during this session, which should all be performed at a rate of 3 to 4 seconds per repetition. A metronome should be utilized to maintain a continuous rate.

The first exercise is a double leg mini squat to a depth of 45° knee flexion. The next exercise is a prone hamstring curl without resistance and later adding a light ankle weight as tolerated. For all exercises, resistance may be progressed when the patient is able to complete all 4 sets of the exercise with appropriate form and speed. Finally, a double leg press initially to a depth of 45° knee flexion, followed by a superset of double leg press heel raises during the rest period. The initial weight used during this exercise series is approximately 30% of the patient’s body weight. The depth of the double leg press and resistance used may be progressed according to patient tolerance.

Phase 3 is postoperative weeks 9 to 12. Before beginning phase 3, patients should achieve full weightbearing, normalized gait pattern, maintain normal joint temperature, and trace to no joint effusion.

The patients begin the BFR session with 1 of the following bridging exercises. Initially, patients perform a double leg bridge. Once tolerated, the patient is progressed to doing single leg bridges. The most challenging progression is a double leg bridge on a ball while maintaining their knees bent, which maximizes hamstring muscle activation.

The second exercise performed is a progression of the squat exercise. The depth of squats should be progressively increased to achieve 90° prior to adding weight. Finally, the patients perform a single leg press and single leg press heel raises during this phase, beginning with 20% of their body weight. The resistance of these exercises is increased throughout phase 3.

Prior to returning to sport, patient’s muscle strength, motion, hop tests, and Y balance tests should be restored to at least 90% of the contralateral side.

The goal of early-stage postoperative meniscus rehabilitation is to minimize the loss of muscle size and strength. When comparing thigh muscle size after 2 weeks of nonweightbearing, application of BFR mitigated the negative changes seen in the control group not receiving BFR.^4^ Furthermore, the BFR group experienced less loss of muscle strength of quadriceps, hamstrings, and plantar flexors with BFR compared with no exercise or with isometric exercise alone. Further studies are warranted to investigate the effects on longer periods of reduced weightbearing.

In weightbearing postoperative populations, studies have demonstrated greater increases in cross-sectional area of quadriceps and hamstring muscles, as assessed by magnetic resonance imaging or ultrasound, in patients whose low-load exercises were supplemented with BFR.^5,10^

Similarly, low-load BFR yields greater muscle strength gains compared with low-load training alone.^3,5^

Comparing low-load BFR training with high-load resistance training yields mixed results. However, it is generally thought that low-load training with BFR may improve muscular strength to a similar extent as high-load strength training, depending on the loading and protocol.^3^ High-load strength training is still recommended to optimize late rehabilitation strength, hypertrophy, and mimic sport-specific demands once safe and well-tolerated.

A systematic review did not reveal significant differences in patient-reported outcome measures.^10^

Several risks have been associated with BFR. The common mild adverse events are increased soreness, subcutaneous hemorrhage (13.1%), and numbness (1.3%). Other mild adverse events reported include pain around the cuff, dizziness, and itchiness.^6,8^ The incidence of severe adverse events is exceedingly low, cumulatively around 0.07% of cases. A recent systematic review and many other studies have shown that BFR training is not more likely to have the adverse events than exercise alone,^6^ indicating that BFR training is safe when applied correctly.

Other downsides to BFR include cost of equipment, time commitment to the therapy, and specialized training for practitioners.

In summary, BFR training may be optimal for patients undergoing meniscal repair as they experience a substantial degree of muscular atrophy. We recommend an automated BFR system inflating to 50% to 80% of total occlusion pressure in combination with low-load resistance exercises. Our rehabilitation protocol includes 3 phases with a frequency of 2 to 3 times per week. Ultimately, BFR training is safe when applied correctly and the rate of serious adverse effects is exceedingly low. Data on its specific effect on meniscal repair outcomes are pending further study.

Here are our references to support our protocol.

Thank you for watching our video.
